# Supplementary material for: Comprehensive analysis of differentially expressed miRNAs in hepatocellular carcinoma: Prognostic, predictive significance and pathway insights
Source: PLoS One. 2024 Apr 18;19(4):e0296198. doi: 10.1371/journal.pone.0296198 (PMC11025735; doi:10.1371/journal.pone.0296198)
Supplement: S1 Table — (DOCX) [file pone.0296198.s001.docx]

**Table S1.** **Number of experimental reads of the predominant mature form of each miRNA.**

| **Previous ID** | **Accession ID** | **Actual IDs** | **Deep sequencing**  **(reads/experiments)** | | **Evidence** | | **Clustered**  **(<10 kb of)** | |
| --- | --- | --- | --- | --- | --- | --- | --- | --- |
|  |  |  | **(5p)** | **(3p)** | |  | |  |
| *hsa-miR-501* | MI0003185 | *hsa-miR-501-5p /*  *hsa-miR-501-3p* | 5759/142 | 48978/153 | | experimental; cloned | | *hsa-mir-532*  *hsa-mir-188*  *hsa-mir-500a*  *hsa-mir-362*  *hsa-mir-501*  *hsa-mir-500b*  *hsa-mir-660*  *hsa-mir-502* |
| *hsa-miR-877* | MI0005561 | *hsa-miR-877-5p /*  *hsa-miR-877-3p* | 13921/149 | 424/69 | | experimental; cloned | |  |
| *hsa-miR-1180* | MI0006273 | *hsa-miR-1180-5p /*  *hsa-miR-1180-3p* | 245/49 | 16487/151 | | experimental; Illumina | |  |
| *hsa-miR-3127* | MI0014144 | *hsa-miR-3127-5p /*  *hsa-miR-3127-3p* | 1220/81 | 47/26 | | experimental; Illumina | |  |
| *hsa-miR-3677* | MI0016078 | *hsa-miR-3677-5p /*  *hsa-miR-3677-3p* | 160/49 | 271/53 | | experimental; Illumina | |  |
| *hsa-miR-99a* | MI0000101 | *hsa-miR-99a-5p /*  *hsa-miR-99a-3p* | 21120938/159 | 29959/149 | | experimental; cloned | |  |
| *hsa-miR-139* | MI0000261 | *hsa-miR-139-5p /*  *hsa-miR-139-3p* | 85265/153 | 21220/136 | | experimental; cloned | |  |
| *hsa-miR-145* | MI0000461 | *hsa-miR-145-5p /*  *hsa-miR-145-3p* | 9052433/150 | 672717/135 | | experimental; cloned | | hsa-mir-143  hsa-mir-145 |
| *hsa-miR-326* | MI0000808 | *hsa-miR-326* | 7980/131 | | experimental; cloned | |  | |
| *hsa-miR-3653* | *hsa-miR-3653* was excluded as the available experimental data no longer supports its annotation as a miRNA (Kozomara et al., 2019) | | | | | | | |
