# Supplementary figures and images for: Comprehensive analysis of differentially expressed miRNAs in hepatocellular carcinoma: Prognostic, predictive significance and pathway insights
Source: PLoS One. 2024 Apr 18;19(4):e0296198. doi: 10.1371/journal.pone.0296198 (PMC11025735; doi:10.1371/journal.pone.0296198)

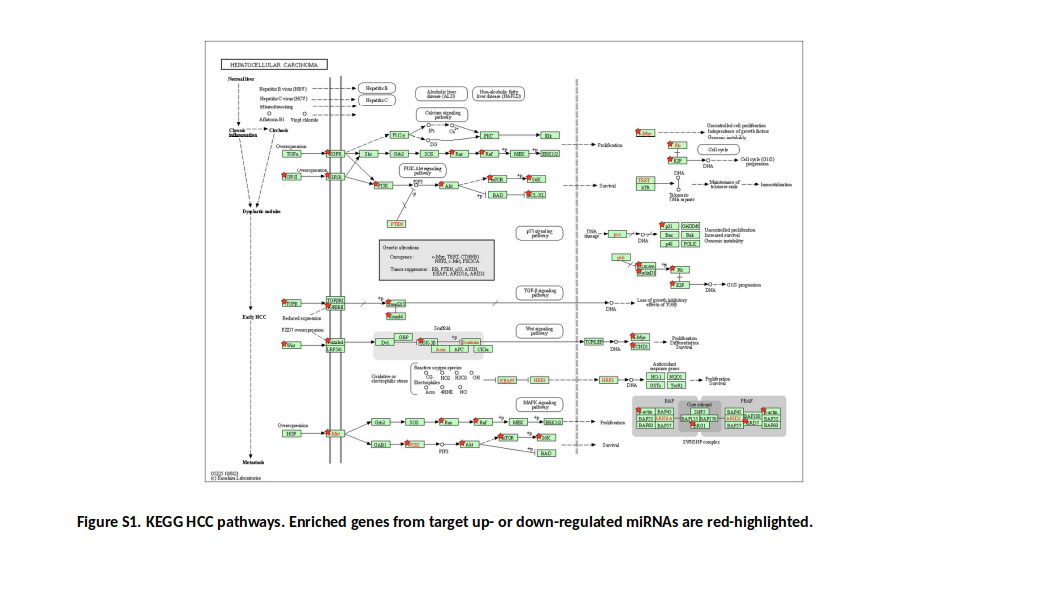

Supplement: S1 Fig — Enriched genes from target up- or down-regulated miRNAs are red-highlighted. (TIF) [file pone.0296198.s003.tif]

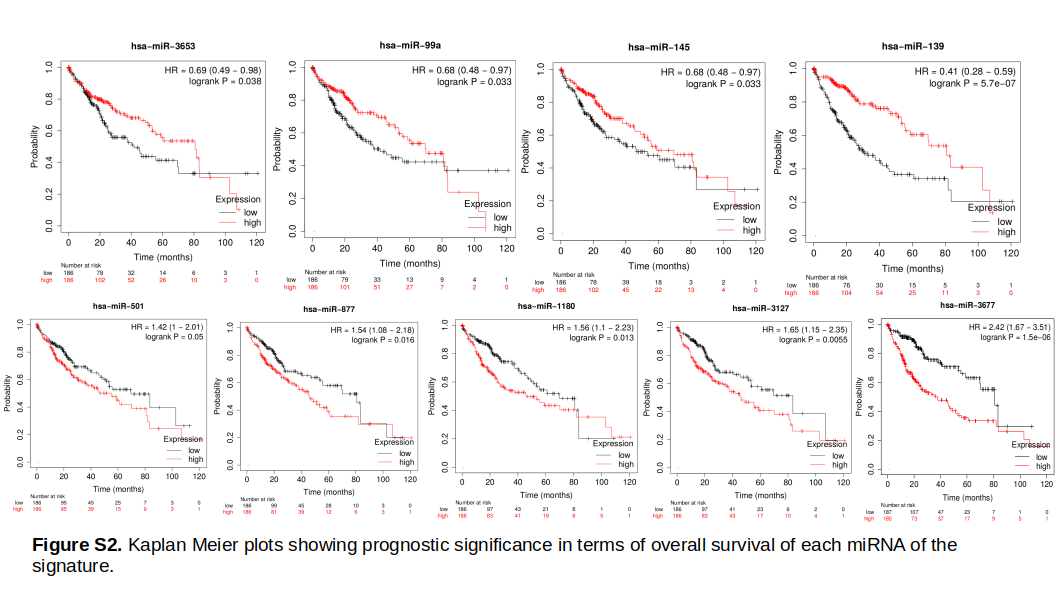

Supplement: S2 Fig — (TIF) [file pone.0296198.s004.tif]
